# Dual electrochemical sensor based on graphene aerogel for quantification of ascorbic acid in pharmaceutical samples and uric acid in urine

**DOI:** 10.1007/s44211-026-00896-z

**Published:** 2026-04-15

**Authors:** Alejandro Gutiérrez, Mario Sánchez-Suárez, Natalia Rey-Raap, Ana Arenillas, Janet Ledesma-García, Luis Gerardo Arriaga

**Affiliations:** 1 División de Investigación y Posgrado, Facultad de Ingeniería, Universidad Autónoma de Querétaro, 76010 Santiago de Querétaro, Mexico; 2 INCAR-CSIC, Instituto de Ciencia y Tecnología del Carbono, Francisco Pintado Fe 26, 33011 Oviedo, Spain; 3 Tecnológico de Monterrey, Institute of Advanced Materials for Sustainable Manufacturing, 76130 Santiago de Querétaro, Mexico

**Keywords:** Graphene aerogel, Uric acid detection, Ascorbic acid detection, Dual electrochemical sensor, Polyethylenimine, Urine

## Abstract

This work presents the advantages of modifying glassy carbon electrodes with a graphene aerogel (GA) dispersed in polyethylenimine (PEI). The presence of GA entrapped in PEI produces a catalytic effect that greatly facilitates the separation of the anodic peaks of ascorbic acid (AA) and uric acid (UA), enabling their detection and quantification by differential pulse voltammetry (DPV). The result is a highly efficient dual electrochemical sensor with a sensitivity of approximately 2.13 × 10^4^ µAM^− 1^ for AA and 5.0 × 10^4^ µAM^− 1^ UA. The current response showed a linear relationship for AA when the concentration ranged from 1.0 to 10 × 10^− 4^ M, with a minimum detection concentration of 0.25 µM and a relative standard deviation (RSD) of 2.0%. For UA, the lineal range was 1.0–15.0 × 10^− 5^ M, with a minimum detection concentration of 9 µM and an RSD of 2.8%. The promising analytical performance, which allows the evaluation of AA and UA, enables accurate quantification of AA in pharmaceutical samples and UA in urine without the use of enzymes or pretreatment.

**Graphical abstract:**

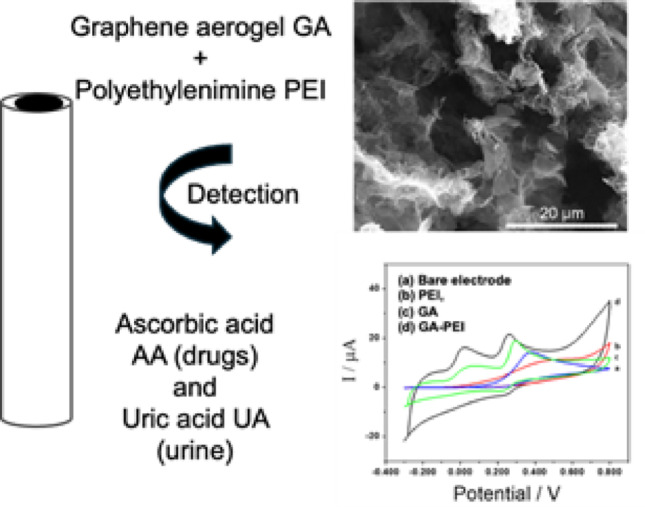

**Supplementary Information:**

The online version contains supplementary material available at 10.1007/s44211-026-00896-z.

## Introduction

In general, ascorbic acid (AA) and uric acid (UA) are both present in biological fluids and play important roles in the physiological metabolic processes of the human body. Therefore, it is necessary to develop sensitive methods for determining of these two biological molecules. Recent research indicates that UA is one of the most important metabolites, as it is the primary end product of purine metabolism in the human body. Excessive accumulation of uric acid can lead to diseases such as gout [1, 2], diabetes [3, 4], cardiovascular disease [2, 4], kidney stones, or renal dysfunction [5–7], so early detection is crucial to prevent these conditions. AA, also known as vitamin C, is a polyhydroxy compound structurally similar to glucose. It affects basic physiological processes and functions in cancer prevention, detoxification, free radical scavenging, and prevention of scurvy [8]. AA is an antioxidant involved in maintaining various neurophysiological processes in the human body, including the synthesis of catecholamines and wound healing [9].

To date, several approaches have proven effective for the individual determination of UA or AA, including chemical and electrochemical luminescence [10, 11], capillary electrophoresis [12], colorimetry and fluorescence [13–15], surface-enhanced Raman scattering [16, 17], liquid chromatography [18, 19], electrochemical sensing, and others [20–22]. However, these techniques are complex to implement, require expensive laboratory equipment, and have long turnaround times. Electroanalytical strategies, such as modified electrodes with suitable electrochemically active materials like polymers, metal nanoparticles, and carbon materials, have shown promise as accurate, simple, and rapid alternatives for the determination of ascorbic acid and uric acid [23–27].

Graphene aerogel (GA) has attracted significant interest due to its unique structure and outstanding properties, including high electron mobility, electrocatalytic activity, large specific surface area, high mechanical strength, and high thermal conductivity [28, 29]. Structurally, GA is highly porous and consists of three-dimensionally connected graphene sheets. Its high specific surface area provides numerous active sites for catalytic reduction processes, and its large pore volume enables rapid transfer of redox species [30–32]. Because of these properties, GA can serve as an ideal support for specific molecules [33–36].

Graphene oxide aerogels have previously been shown to be effective in developing sensors for cancer cells, organic compounds, H_2_O_2_, pollutants, and others targets [35–43].

In this work, a competitive and low-cost electrochemical sensor based on PEI and a graphene aerogel, produced through a fast, simple, and scalable process, is proposed. The ratio of the components (GA and PEI) and the mixing method were optimized based on the electrochemical response to AA and UA. The analytical application of glassy carbon electrodes (GCEs) modified with the resulting dispersion (GCE/GA-PEI) was evaluated for the sensitive and selective quantification of AA (or UA) in the presence of UA (or AA, respectively). The promising dual sensor demonstrated good analytical performance, with low detection limits and high selectivity, even in complex matrices such as urine, which was used without pretreatment, and in commercial pharmaceutical samples without the need for enzymes.

## Experimental section

### Reagents and materials

Resorcinol was purchased from Sumitomo Chemical Co. Ltd., and the graphene oxide dispersion (5 mg ml^− 1^) from ApplyNanoSolution S.L. Ascorbic acid was purchased from MACRON Fine Chemical. A 37 wt% formaldehyde solution, uric acid and polyethylenimine (PEI, MW 750,000, catalog number 25414-2) were purchased from Sigma-Aldrich. Ethanol was obtained from Karal. All chemicals were of reagent grade and used without further purification.

A 0.050 M phosphate buffer solution at pH 7.40 was used as the supporting electrolyte. Ultrapure water (ρ = 5 MΩ cm^− 1^) from the Millipore-MilliQ system was used to prepare of all solutions and dispersions.

### Synthesis of graphene aerogel

6.5 g of resorcinol was dissolved in 186.8 g of graphene oxide solution with stirring for 30 min to ensure complete dissolution. Then, 8.8 g of formaldehyde solution was added, and the mixture was stirred further to achieve homogeneity. NaOH was added dropwise with constant stirring to adjust the pH to 5.0. This precursor mixture was heated in a glass beaker at 85 °C under microwave radiation for 3 h to complete the sol–gel reaction and curing phase [44, 45]. The material was then cooled to room temperature and frozen with liquid nitrogen. It was dried by sublimation of water in a freeze dryer (HyperCOOL HC3110, Gyrozen Co., Ltd.) at − 110 °C for 2 days under vacuum. Once dried, the material was carbonized in a tube furnace at 1000 °C for 2 h under a nitrogen atmosphere to obtain the final graphene aerogel (GA). Finally, the material was ground in a ball mill to obtain a powder with a particle size of less than 75 microns.

### Physico-chemical and electrochemical characterization

The morphology of the samples was analyzed using a Quanta FEG 650 (FEI Company, Hillsboro) scanning electron microscope (SEM) equipped with an Everhart–Thornley detector.

The porous properties of GA were evaluated by N_2_ adsorption-desorption isotherms at 196 °C (Micromeritics Tristar II). From these isotherms, textural parameters such as specific surface area (S_BET_) and external surface area (S_ext_) were determined using the Brunauer-Emmett-Teller equation and the t-plot method, respectively. The pore volume (V_p_) was determined from the total amount of adsorbed nitrogen at the saturation point (p/p^0^ = 0.99). The percentage of porosity (Ɛ) and total pore volume (V_T_) were measured with a Geopyc 1365 instrument (Micromeritics). The sample was degassed overnight at 120 °C before each analysis.

The electrical conductivity (K) of the graphene aerogel was determined using the four-probe method, in which the resistivity of disk-shaped GA pellets was measured with an Everbeing SR-4–6 L four-point prober, a Keithley Model 62,220 DC power source, and a Keithley Model 2182 A digital nanovoltmeter. The pellets were prepared by mixing 90 wt% GA with 10 wt% polytetrafluoroethylene (PTFE, Sigma-Aldrich) in absolute ethanol until a homogeneous paste formed, from which the pellets were die-cut. The die-cut pellets were uniaxially pressed at 10 t for 30 s to ensure good particle contact, then dried overnight at 60 °C. After pressing, the pellets measured 1 cm in diameter and 0.2 mm in thickness. Electrical conductivity measurements were performed on three different pellets from the same sample, with six measurements taken on each pellet.

X-ray photoelectron spectroscopy (XPS) was performed using a Kratos AXIS Ultra HAS with a monochromatic Al Kα X-ray source (1486.7 eV), using a pass energy of 100 eV for survey scans and 30 eV for high-resolution regions. Data were analyzed with CasaXPS, applying the Shirley background. For peak deconvolution, a Gaussian-Lorentzian shape was used for all peaks except the carbon sp2 peak, which was fitted with an asymmetric peak shape.

Electrochemical experiments were conducted using a Biologic and an Epsilon (BAS) potentiostat. An Ag/AgCl, 3 M NaCl (BAS, model RE-5B) electrode and a platinum wire served as the reference and counter electrodes, respectively. All potentials are referenced to the Ag/AgCl electrode. Bare glassy carbon electrodes (GCE) and GCEs modified with GA, PEI and GA-PEI were tested as working electrodes. A magnetic stirrer (BASi Cell stand) and a stir bar provided convective transport during amperometric measurements at 600 rpm.

### Electrochemical impedance spectroscopy (EIS)

The electronic properties of the different surfaces were characterized by EIS. The experiments were performed by applying a sinusoidal potential perturbation with an amplitude of 10 mV in the frequency range of 10^5^–10^− 1^ Hz using 2.50 × 10^− 2^ M H_2_O_2_ as a redox marker to evaluate surface blocking and the effects of surface charges in the system.

### Electrochemical sensor

The glassy carbon electrode (GCE) was first polished with aluminum oxide (1.0, 0.30 and 0.050 μm) for 2 min with each particle size. The electrode was then thoroughly rinsed with deionized water and sonicated in water for 5 s. Next, the GCE was cycled in a 0.050 M phosphate buffer solution at pH 7.40 from − 0.300 to 0.800 V at a scan rate of 0.050 V s^− 1^ for 10 cycles, then cleaned with ultrapure water and dried with N_2_.

To modify the GCE with PEI (GCE/PEI), a 20 µL drop of 1.0 mg mL^−1^ PEI solution (50:50 v/v water: ethanol) was placed on the GCE surface. For GCE/GA (GCE modified with graphene aerogel (GA)), a 20 µL drop of GA dispersion was applied to the GCE surface. The GA dispersion was prepared by sonicating 0.5 mg GA in 1.0 mL of a mixed solution (50:50 v/v water: ethanol) for 15 min. Glassy carbon electrodes modified with both GA and PEI (GCE/GA-PEI) were prepared by applying 20 µL of the GA-PEI dispersion onto the polished GCE and drying for 50 min. The GA-PEI dispersion was obtained by sonicating 0.5 mg GA in 1.0 mL of a 1.0 mg mL^−1^ PEI solution (50:50 v/v water: ethanol) for 15 min, followed by centrifugation for 15 min at 1000 rpm. The supernatant was collected for further use.

The modified GCE electrodes were cycled in phosphate buffer solution at pH 7.40 between − 0.300 and 0.800 V at 0.050 V s^−1^ for 5 cycles.

AA and UA were quantified by differential pulse voltammetry (DPV) by scanning the potential from − 0.300 V to 0.800 V at 0.050 V s^−1^. The analytical signal was obtained from the oxidation peak current of AA and UA after subtracting the background current. All measurements were performed at room temperature.

The electrochemical uric acid sensor was validated using the Uricostat enzymatic kit from Wiener Lab. (Mexico), a reference method for quantifying UA in clinical laboratories [46, 47]. This method relies on the uricase-catalyzed conversion of UA to allantoin and oxygen. In a second step, the enzymatically generated hydrogen peroxide, in the presence of peroxidase, 4-aminophenazone, and dichlorohydroxybenzenesulfonic acid, forms quinonimine, a compound that absorbs at 505 nm. The urine sample was homogenized for 24 h and used untreated for UA quantification. The standard calibration curve method was used for detecting UA in urine or AA in drugs.

## Results and discussion

### Physico-chemical properties of the graphene aerogel

**Fig. 1:**
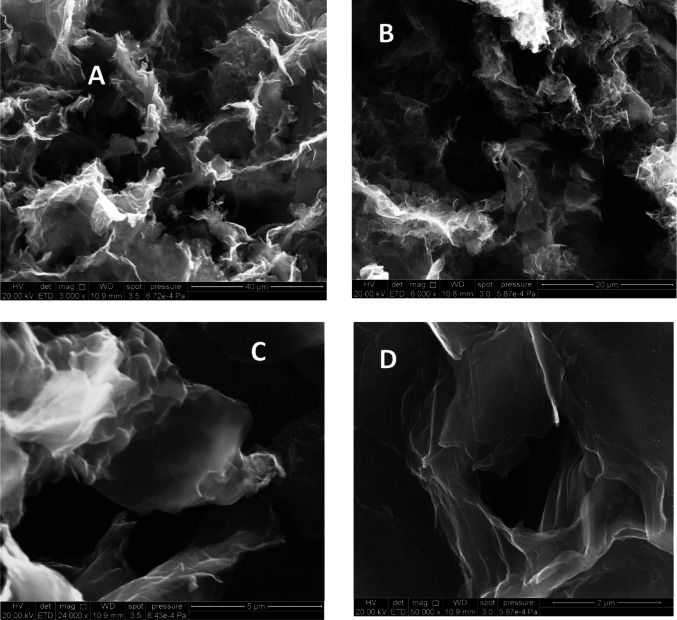
SEM images of the graphene aerogel obtained at different magnifications: **A** 3000×, **B** 6000×, **C** 24.000× and **D** 50,000×

The morphology of the synthesized GA was examined by SEM. Images at different magnifications are shown in Fig. 1.

GA has an architecture composed of randomly distributed, interconnected, thin, crimped graphene sheets with pore sizes of several micrometers (Fig. 1), reinforced by amorphous carbon from the RF polymer. The SEM image of dispersion GA–PEI shows a homogeneous morphology and well-distributed interconnections between the graphene and polymer (image not shown). The interconnections between the sheets are formed by mechanisms such as van der Waals attractions, π–π stacking of the remaining sp2-hybridized regions in the basal plane [29], and bonds created by the carbonized RF polymer. These features provide mechanical strength to the 3D scaffold [45] and enable the formation of a highly porous structure with low density and large pore volume (Table 1).

**Table 1:** Physicochemical properties of the synthesized graphene aerogel

Sample	Ɛ (%)	V_T_ (cm^3^g^− 1^)	K (S m^− 1^)	S_BET_ (m^2^g^− 1^)	S_ext_ (m^2^g^− 1^)	V_*p*_ (cm^3^g^− 1^)
GA	93	6.7	700	204	27	0.16

Despite its highly porous structure, GA exhibits high electrical conductivity because the continuous and well-distributed interconnections between the graphene layers facilitate electron transfer (Table 1). This combination of properties in a single material is unusual, as porosity (structural and/or void) and electrical conductivity are typically opposing characteristics, although both are essential for electrochemical sensors. GA shows porosity of about 90% and high conductivity of 700 S m^−1^.

**Fig. 2:**
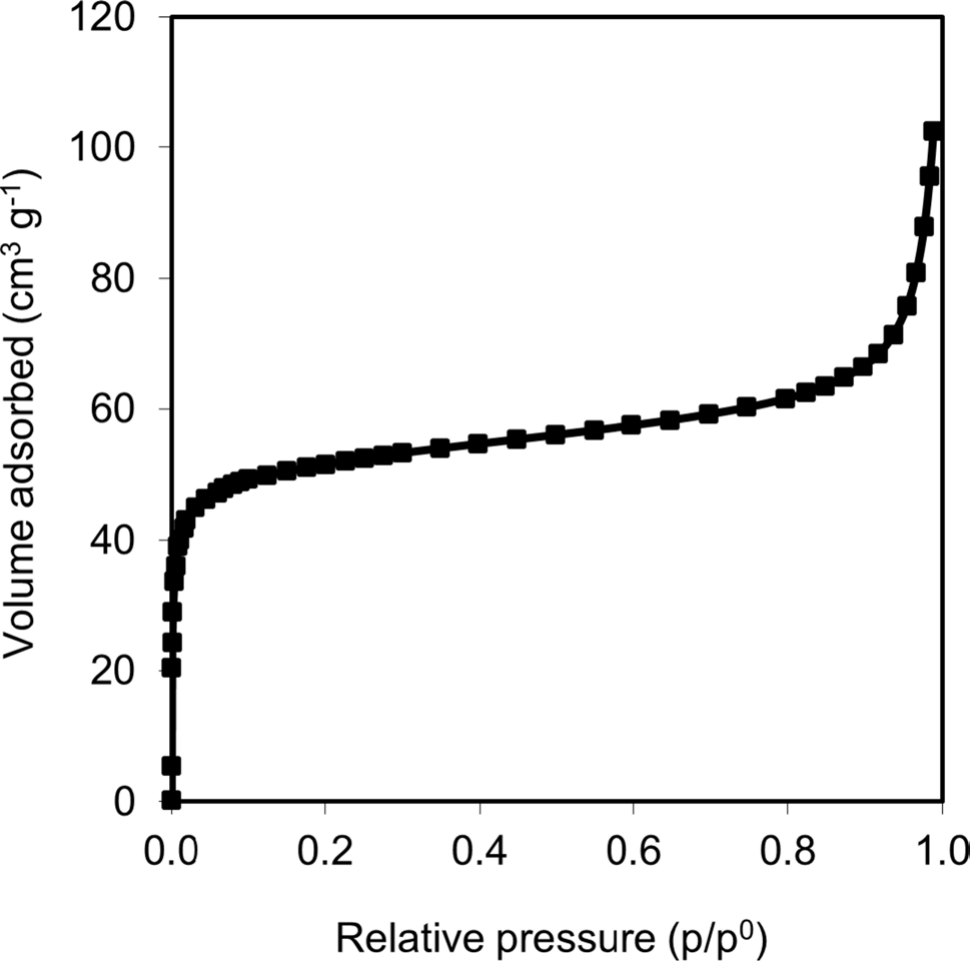
Nitrogen adsorption isotherm of the synthesized graphene aerogel

Figure 2 shows the nitrogen adsorption isotherm of the GA sample, which corresponds to a combination of type I and type II isotherms according to the IUPAC classification. At low relative pressures, the behavior resembles type I isotherms due to the high adsorbed volume, indicating the presence of micropores and a large specific surface area, as described in Table 1. At high relative pressures, the shape of the isotherm can be correlated with a type II isotherm, characteristic of macroporous materials, which is consistent with the SEM images in Fig. 1. Additionally, the external surface area is less than 30 m^2^ g^−1^, which is typical of macroporous aerogels [48]. This macroporosity gives the material a very high total pore volume (VT) and a high percentage of porosity (93%), as shown in Table 1.

**Fig. 3:**
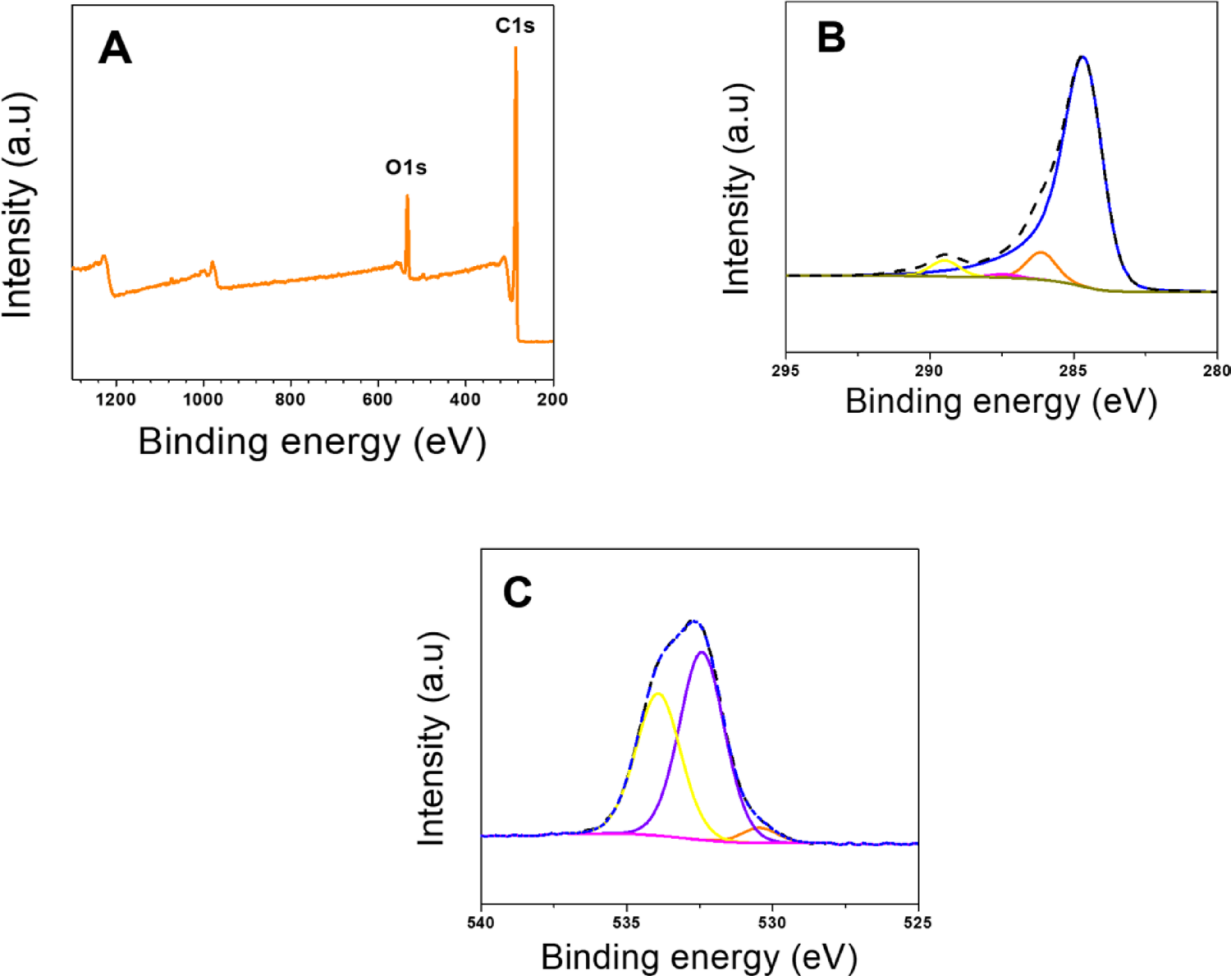
High-resolution XPS general spectra for **A** GA aerogel, **B** C 1s region, and **C** O 1s region

The surface elemental composition and the nature of the oxygen functional groups in the graphene aerogel were analyzed by XPS. According to the survey spectra (Fig. 3A), the sample consists of 91.4 at% carbon and 8.6 at% oxygen. The high-resolution XPS spectrum in the C 1s region was deconvoluted into five main peaks (Fig. 3B) corresponding to C = C (284.5 eV), C–OH (286.1 eV), C=O (287.4 eV), COOH (289.5 eV), and the shake-up satellite due to π–π∗ transitions in aromatic rings (290.9 eV). The contribution of each oxygen group suggests that the graphene aerogel is mainly composed of alcohol or phenol bonds and carboxyl groups, indicating that its surface is acidic. This trend in oxygen functional groups is also observed in the deconvolution of the high-resolution spectrum in the O 1s region (Fig. 3C).

### Optimization and characterization of the electrochemical sensor electrode

**Fig. 4:**
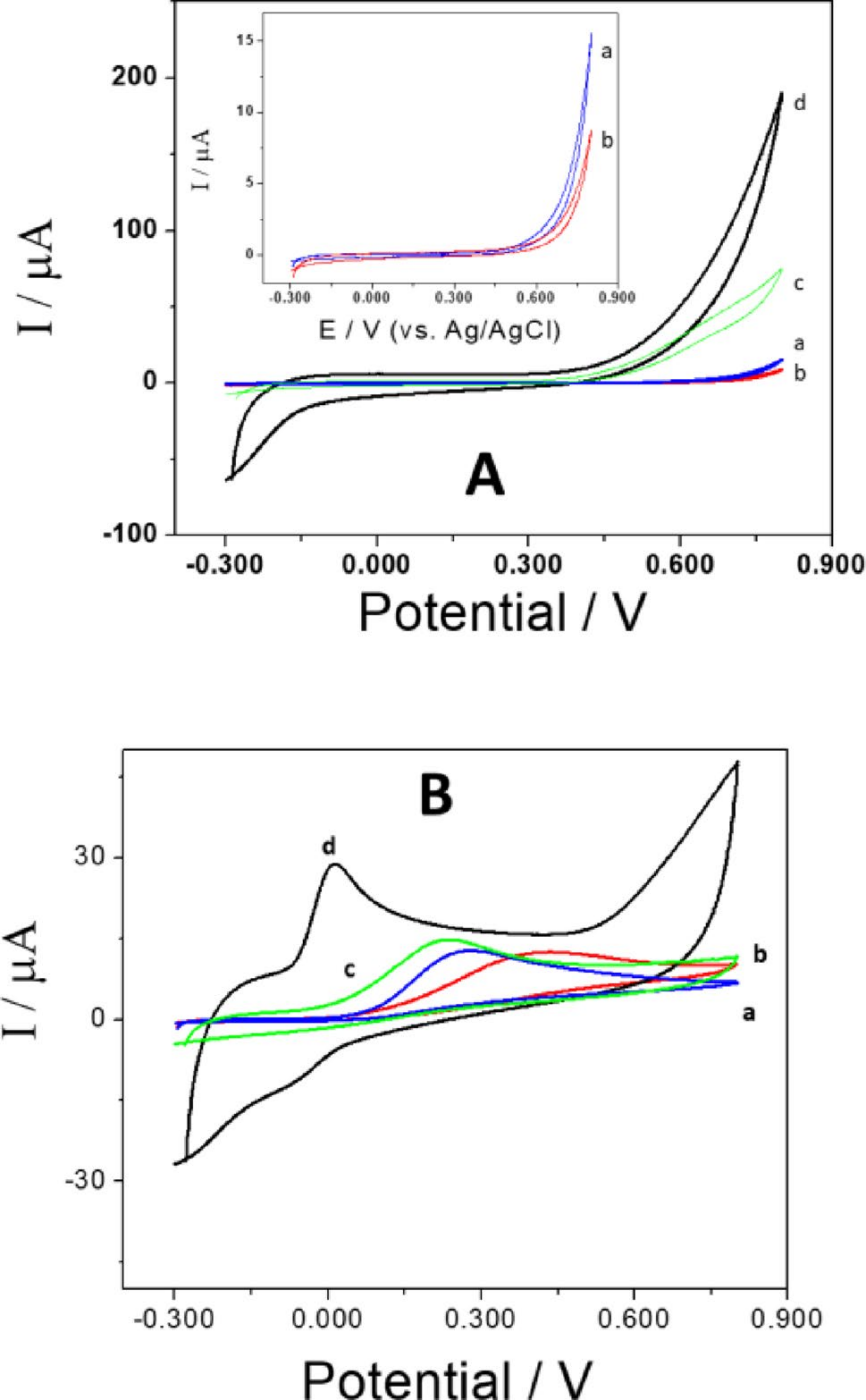
Cyclic voltammograms for 5.0 × 10^−2^ M hydrogen peroxide (**A**) and 1.0 × 10^−3^ M AA (**B**), using GCE (a), GCE/PEI (b), GCE/GA (c), and GCE/GA-PEI (d). In all cases, 0.050 M phosphate buffer solution at pH 7.40 was used as the electrolyte, with a scan rate of 0.050 V s^−1^

Figure 4A shows cyclic voltammograms for 50 mM H_2_O_2_ at GCE (a), GCE/PEI (b), GCE/GA (c) and GCE/GA-PEI (d). The voltammetric response at GCE/GA-PEI (0.700 V) for hydrogen peroxide shows a remarkable increase in oxidation/reduction current: twenty times higher than GCE, forty times higher than GCE/PEI, and twice as high as GCE/GA. The potentiodynamic profiles for a 1.0 mM AA solution with GCE/GA-PEI show a reduction in oxidation overpotential by 0.300 V, 0.455 V, and 0.193 V compared to GCE, GCE/PEI, and GCE/GA, respectively, due to the presence of positive and negative charges (polymer and anion). The oxidation current was significantly increased, directly related to the electroactive area of GA. This results from the combined effect of GA and PEI on catalytic activity, attributed to the interaction of the positively charged GA-PEI dispersion, the unhindered diffusion of compounds in the highly porous platform, and the good electrical conductivity (Fig. 4B). In Fig. 4, two different species (H_2_O_2_ and ascorbic acid), neutral and negative respectively, were used; these studies were conducted to enable new possibilities for developing enzyme electrochemical sensors for the determination of H_2_O_2_.

Additionally, Figure S1A (Supplementary Material) presents Nyquist plots obtained in the presence of 2.50 × 10^−2^ M hydrogen peroxide at 0.700 V for (a) GCE, (b) GCE/PEI, (c) GCE/GA, and (d) GCE/GA-PEI. The experimental data were satisfactorily fitted with the simple equivalent circuit R_s_(R_ct_C_dl_). The components represent R_ct_, the charge transfer resistance, which is in parallel with C_dl_, the double layer capacitance; both are in series with R_s_, the electrolytic resistance. Figure S1B shows the changes in R_ct_ and C_dl_. It is clear that R_ct_ increases when GCE is modified with PEI, due to surface blocking (22,000 and 27,000 Ω for GCE and GCE/PEI, respectively). The passivation effect is evident when comparing the Nyquist plots for GCE/GA and GCE/GA-PEI (1100 and 1800 Ω, respectively). This indicates that the large area of GA allows a significant increase in the charge transfer rate of the redox producer, even though the PEI supporting GA has a passivating effect.

A crucial aspect in developing GA-based electrochemical sensors is finding a strategy that enables stable and effective functionalization of the nanostructures, as well as their accurate dispersion and deposition on the electrode surface in a robust and homogeneous manner.

In this work, the influence of GA amount, PEI concentration, and sonication time was investigated to quantify dispersion efficiency by amperometry and cyclic voltammetry, using hydrogen peroxide and AA as redox markers, respectively. These redox markers are different species: H_2_O_2_ at pH 7.4 is a neutral molecule, while AA is a negatively charged molecule.

**Fig. 5:**
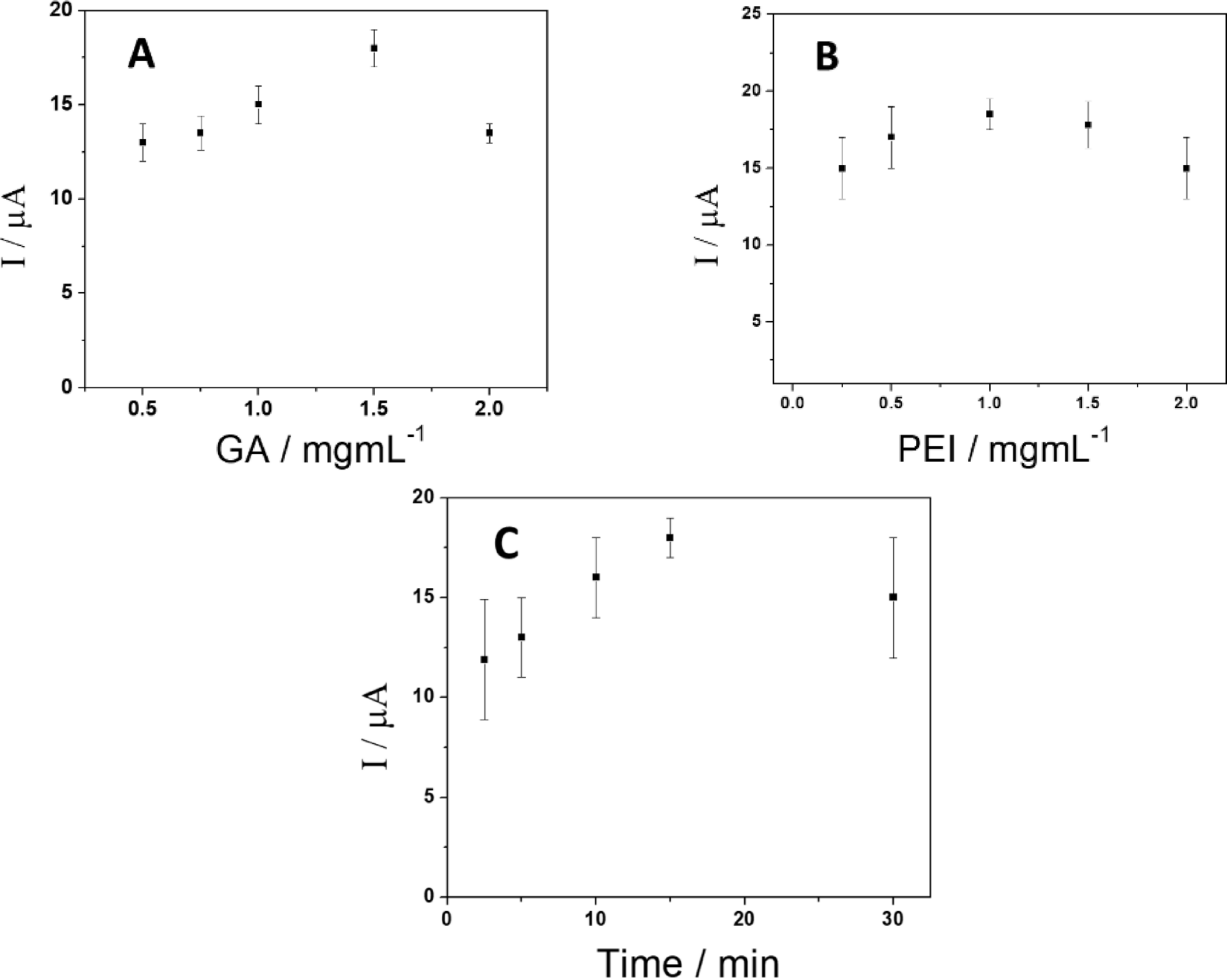
Variation of peak current during oxidation of ascorbic acid by CV using **A** GCE/GA-PEI prepared with 15 min of sonication, 1.0 mg mL^− 1^ PEI, and different amounts of GA; **B** GCE/GA-PEI produced by 15 min of sonication, 1.5 mg mL^− 1^ GA and different concentrations of PEI; **C** as a function of sonication time, using 1.5 mg mL^− 1^ GA with 1.0 mg mL^− 1^ PEI. The error bars represent the standard error of the mean (*n* = 3 electrodes)

The effect of GA amount was examined using 1.0 mg mL^− 1^ PEI (Fig. 5A) and 1.0 mM AA as a redox marker. The oxidation current for AA increases as the amount of GA increases from 0.5 to 1.5 mg mL^− 1^, which is attributed to the electroactivity of the nanostructure. However, higher amounts of GA in the dispersion result in a decrease in the reaction due to less efficient dispersion at this PEI concentration. Therefore, 1.5 mg mL^− 1^ GA was selected as the optimal amount of carbon nanostructures.

The ratio of PEI to GA was analyzed in the presence of AA. Figure 5B shows the effect of PEI concentration on the current for AA oxidation using a GCE modified with a dispersion of GA (1.5 mg mL^−1^) in PEI solutions of varying concentrations (0.25–2.0 mg mL^−1^). The oxidation current increases when the GCE is modified with a GA-PEI dispersion prepared with 0.25 mg mL^−1^, 0.50 mg mL^−1^, or 1.0 mg mL^−1^ PEI, demonstrating the efficiency of PEI as a dispersant. Dispersions prepared with 1.5 and 2.0 mg mL^−1^ PEI resulted in decreased response due to the passivation effect of the polymer at higher concentrations. Therefore, 1.0 mg mL^−1^ PEI was suggested as the optimal amount in an ethanol: water (1:1) solution with 1.5 mg mL^−1^ GA.

An important factor in preparing GA polymer dispersions, is the sonication time, as the total energy supplied by the ultrasonic process alters the sequence of cavitation events that facilitate the disruption of nanostructures. The effect of sonication time (between 2.5 and 30 min) on the peak current for AA oxidation is shown in Fig. 5C. The signal increases by approximately 6 µA as the sonication time increases from 2.5 to 15 min, with no further increase at longer times.

The results show that PEI is an excellent dispersant for GA, and optimal dispersion is achieved by mixing 1.5 mg mL^− 1^ GA with 1.0 mg mL^− 1^ PEI (prepared in an ethanol: water solution) and sonicating for 15 min.

Other similar experiments also confirmed the benefits of this dispersion on sensitivity to hydrogen peroxide. Figure S2 shows the variation in sensitivity to hydrogen peroxide from amperometric experiments performed at 0.700 V on GCEs modified with (A) different amounts of GA and 1.0 mg mL^− 1^ PEI and (B) 1.5 mg mL^− 1^ GA and different concentrations of PEI. A large improvement in sensitivity is achieved when the amount of GA increases from 0.25 to 1.5 mg mL^− 1^. However, 2.0 mg mL^− 1^ GA impairs the quality of the dispersion and increases the uncertainty. Figure S2 (B) shows how the sensitivity increases when GCE is modified with dispersions prepared with 0.25 mg mL^− 1^, 0.50 mg mL^− 1^or 1.0 mg mL^− 1^ PEI. For dispersions prepared with 1.5 and 2.0 mg mL^− 1^ PEI, sensitivity decreases due to the passivating effect.

The stability of the GA-PEI dispersion is important for further applications in electrochemical sensors. Figure S3 shows the sensitivity to the redox marker (hydrogen peroxide) with the best dispersion, which was stored at 294 K. The results were obtained at 0.700 V with different GCEs modified with the same GA-PEI dispersion on different days. No significant changes were observed even after 30 days of storage, with the sensitivity remaining at 95% of the original value.

### Electrochemical detection of ascorbic acid and uric acid

**Fig. 6:**
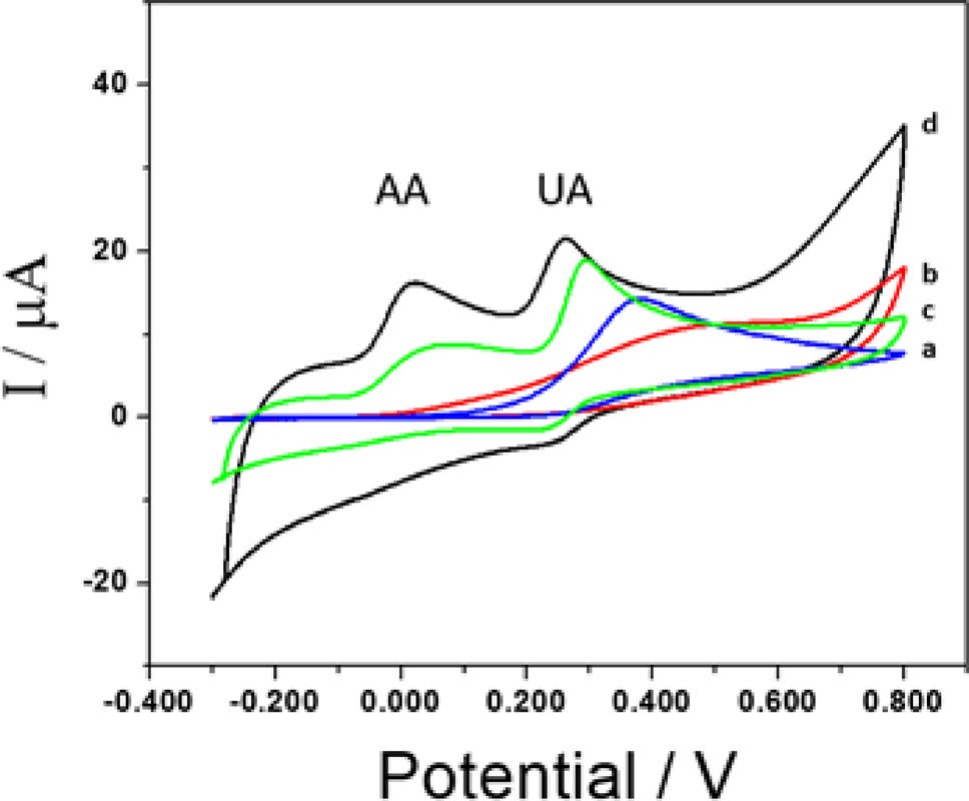
Cyclic voltammetry of 0.5 × 10^−3^ M AA and 0.5 × 10^−3^ M UA on **a** GCE, **b** GCE/PEI, GCE/GA, and **c** GCE/GA-PEI. In all cases, a 0.050 M phosphate buffer solution at pH 7.40 was used as the electrolyte, with a scan rate of 0.050 V s^−1^

Figure 6 shows irreversible oxidation on bare GCE. The oxidation peak appears at 0.42 ± 0.01 V, and the oxidation current is 12 ± 1 µA, consistent with other published work [49]. GCE/PEI also exhibits only an irreversible oxidation peak at 0.56 ± 0.01 V with a current of 10 ± 1 µA. When the profile is obtained with GCE/GA-PEI (and GCE/GA), two oxidation peaks are detected: one corresponding to AA at 0.015 ± 0.007 V (and 0.089 ± 0.005 V) with a peak current of 8.3 ± 0.5 µA (and 5.8 ± 0.9 µA) due to the oxidation of ribose, and another corresponding to the oxidation of purine from UA at 0.259 ± 0.007 V (and 0.296 ± 0.005 V) with a current of 8.6 ± 0.5 µA (and 11.0 ± 0.9 µA). The GCE/GA electrode is unstable and not reproducible. ΔE_AA−UA_ = 0.244 V (and ΔE_AA−UA_ = 0.207 V).

**Fig. 7:**
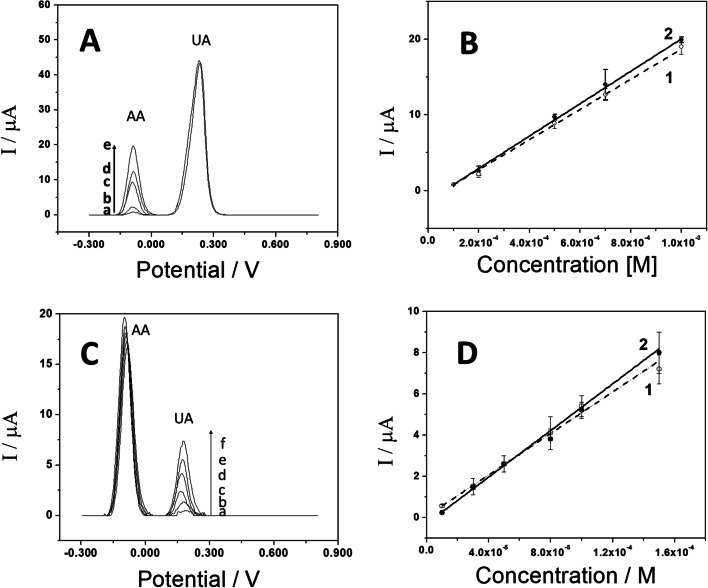
Differential pulse voltammograms of GCE/GA-PEI obtained in 0.050 M phosphate buffer solution (pH 7.40) containing (**A**) 1.0 × 10^−3^ M UA with AA in the concentration range of 1.0 × 10^−4^ to 1.0 × 10^−3^ M, and (**C**) 1.0 × 10^−3^ M AA with UA in the concentration range of 1.0 × 10^−5^ to 1.5 × 10^−4^ M. Calibration plot for AA (**B**) in the absence (open circles) and presence (solid circles) of 1.0 × 10^−3^ M UA, and for UA (**D**) in the presence (open circles) and absence (solid circles) of 1.0 × 10^−3^ M AA. Error bars indicate the standard error of the mean (*n* = 3 electrodes)

Given the advantage of using the GCE/GA-PEI, this modified working electrode was employed to detect AA and/or UA by differential pulse voltammetry (DPV) to minimize the influence of background current. The peak oxidation current was proportional to the concentration of AA, and a linear relationship was observed in the range of 0.1 mM to 1.0 mM (Figure S4A). Figure 7A presents differential pulse voltammograms obtained in a 0.050 M phosphate buffer solution at pH 7.40 for (a) 1.0 × 10^−4^ M, (b) 2.0 × 10^−4^ M, (c) 5.0 × 10^−4^ M, (d) 7.0 × 10^−4^ M, and (e) 1.0 × 10^−3^ M AA in the presence of 1.0 × 10^−3^ M UA. The oxidation peaks at − 0.096 V and 0.200 V correspond to the oxidation of AA and UA, respectively, with ΔE_AA−UA_ = 0.296 V.

Figure 7B presents the calibration plot for AA in the absence (open circles) and presence (filled circles) of 1.0 × 10^−3^ M UA. The sensitivity for AA without UA was (2.13 ± 0.04) µA M^−1^ (*r* = 0.9994), with a linear range from 1.0 × 10^−4^ to 1.0 × 10^−3^ M, and a limit of detection of 0.25 µM (calculated as 3.3 × σ/s, where σ is the standard deviation of the blank signal and s is the sensitivity). The calibration plot for AA in the presence of 1.0 × 10^−3^ M UA showed a similar linear range in the absence of UA. The sensitivity was (1.99 ± 0.07) µA M^−1^ (*r* = 0.9958), which is 6.9% lower than that obtained without UA. The electrochemical sensor had a limit of detection of 0.27 µM, demonstrating its effectiveness for quantifying AA in the presence of UA, its most competitive metabolite. Each measurement was performed with a new electrode, and each test was conducted in triplicate. The relative standard deviation (R.S.D.) for determining 5.0 × 10^−4^ M AA using eight different electrodes modified with the same dispersion was 0.9%. The R.S.D. for determining 5.0 × 10^−4^ M AA using ten different dispersions was 2.0%.

UA determination was performed in the same manner. Figure 7C shows DPVs obtained in a 0.050 M phosphate buffer solution at pH 7.40 for (a) 1.0 × 10^−5^ M, (b) 3.0 × 10^−5^ M, (c) 5.0 × 10^−5^ M, (d) 8.0 × 10^−5^ M, (e) 1.0 × 10^−4^ M, and (f) 1.5 × 10^−4^ M UA in the presence of 1.0 × 10^−3^ M AA. The first oxidation peak at (− 0.091 ± 0.004) V is due to AA, and the second oxidation peak is due to UA. Figure S4B shows the DPVs under the same conditions as in Fig. 7C, but without 1.0 × 10^−3^ M AA. Figure 7D displays the calibration plot for UA in the presence (open circles) and absence (solid circles) of 1.0 × 10^−3^ M AA. The sensitivity for UA in the absence of AA was (5.0 ± 0.3) × 10⁴ µA M^−1^ (*r* = 0.9956), with a linear range between 1.0 × 10^−3^ and 1.5 × 10^−4^ M, and a limit of detection of 10 µM UA. In the presence of 1.0 × 10^−3^ M AA, the sensitivity was (5.6 ± 0.2) × 10⁴ µA M^−1^ (*r* = 0.9933), and the limit of detection was 9 µM UA, showing a correlation between the sensitivities of the two calibration plots. Each experimental point represents the average of three determinations performed with three different electrodes. The R.S.D. for the determination of 5.0 × 10^−5^ M UA with five different electrodes modified with the same dispersion was 1.5%. The R.S.D. for the determination of 5.0 × 10^−5^ M UA using seven different dispersions was 2.8%.

### Determination of AA and UA in complex real mixtures

The GCE/PEI-GA sensor was used to analyze various drugs, including Redoxon Kids from Bayer, Noratoquim from Quimpharma, and Aderogil^®^, all of which are popular drugs containing AA. Measurements were performed by DPV in triplicate. Table 2 summarizes the AA concentrations detected with the GCE/PEI-GA sensor and the values reported on the drug labels. The recovery rates for the different AA samples ranged from 99.7 to 101%. Excellent correlation was achieved, with error values between 0.1 and 1%.

**Table 2:** Quantification of AA using GCE/GA-PEI as the working electrode in the electrochemical sensor for different types of commercial drugs

Analyte		Label content (mg)	Experimental determination with GCE/GA-PEI	RSD (%)	Error (%)
AA	Redoxon	100	101 ± 1	2.5	1
	Noratoquim	1000	997 ± 3	4.2	0.3
	Aderogyl^®^	1000	999 ± 4	5.2	0.1

**Fig. 8:**
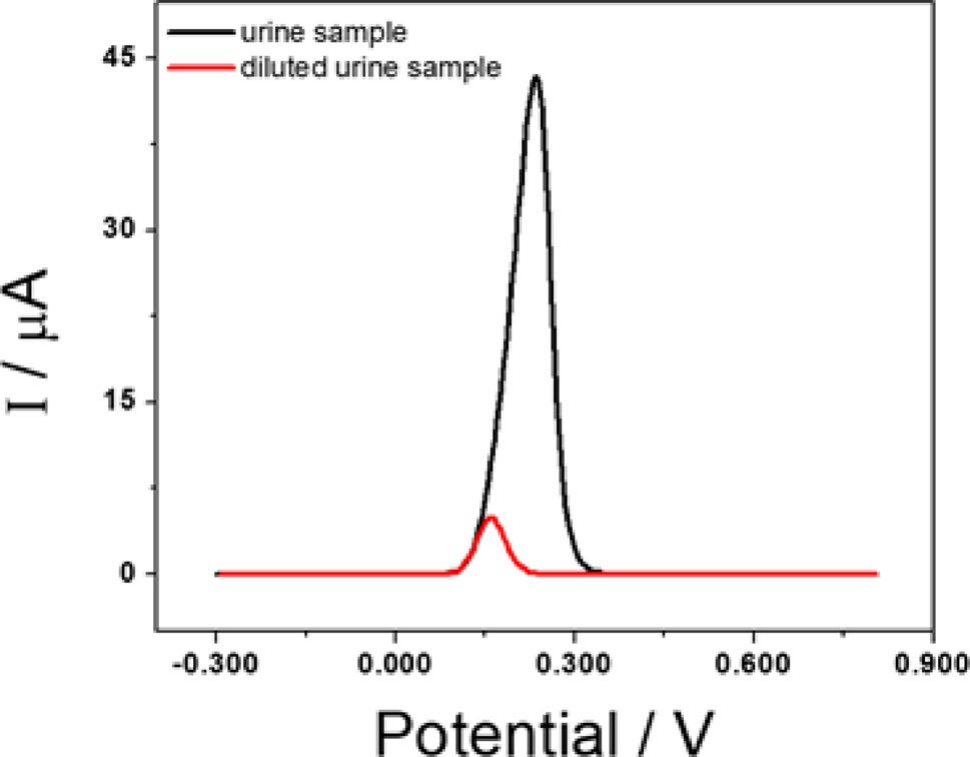
Differential pulse voltammograms of GCE/GA-PEI obtained in a 0.050 M phosphate buffer solution (pH 7.40) containing a urine sample (black) and a 60-fold diluted urine sample (red)

The GCE/GA-PEI sensor was also used to quantify UA in urine without pretreatment. Figure 8 shows the results of urine sample experiments during electrochemical investigation. Urine is a complex matrix containing interfering electroactive compounds such as glucose, urea, lactic acid, K^+^, Na^+^, Cl^−^, NO_2_^−^, albumin and creatinine [50]. However, in the voltammograms, only one peak appeared, which was attributed to UA oxidation.

For the analytical test, the urine was diluted 60-fold with 0.050 M phosphate buffer at pH 7.40. The concentration of UA in the urine sample determined with the proposed sensor was (16 ± 0.4) mg mL^− 1^ (average of ten determinations), which agrees with the value obtained by the enzymatic method with spectrophotometric quantification (16 ± 0.2) mg mL^− 1^. The percentage recovery after addition of 1.0 × 10^− 4^ M UA to the diluted urine sample was evaluated, with values ranging from 97 to 104%, demonstrating excellent efficiency.

Table 3 compares the analytical performance of our AA and UA sensor based on nanomaterials reported in recent years. Compared with most relevant sensors, our sensor has a better limit of detection than those in [10, 22, 24, 49, 50] and is comparable to those in [12, 21, 51]. Generally, sensors with better limits of detection than ours are composed of multiple components, which can make fabrication more time-consuming [21, 24, 52]. In summary, the analytical platform proposed here provides sensitive and selective detection of AA in the presence of UA, and vice versa, in a very simple manner, without GA functionalization or the addition of polymeric barriers or metal nanoparticles. These aspects make it a highly competitive and useful strategy for selective detection.

**Table 3:** Comparison of ascorbic acid and uric acid detection using different sensor platforms

Sensing platform	Target molecule	Technique	Linear range (µM)	LOD (µM)	Ref.
N-doped graphene aerogel	AA	SWV	100–1000	0.06	[9]
[Co(HL) (bpe)] (H3L ¼ 5-(3,4- dicarboxylphenoxy) nicotic acid; bpe ¼ 1,2-bis(4-pyridyl) ethylene)	AA	Luminescence turn-off	0–25	0.68	[10]
HP^3D^CE	UA	Spectrometric measurement	1.68–117.6	5.4	[12]
PEDOT: PSS/PVA/AuNPs/SPCE	UA	DPV	0–1000	2.99	[20]
γ-Ni(OH)_2_–NiO(OH)/PANI-CNTs	UA	DPV	5–100 100–800	0.11	[21]
CUST-636 CUST-638	AA	VC	0–8000		[22]
TMB-Pt@Ag NFs/uricase	UA	colorimetry	0.5–150	0.3	[24]
MWCNT-polyArg	AA	DPV	6–70	0.95	[49]
Pd-Co / UOx Ni-Co / UOx	UA UA	amperometry	0–250 0–100	18 14.6	[50]
WB-S La^3+^/TiO_2_- NS/SPE	AA	DPV	0.001–900	0.00063	[52]
LIG	UA	DPV	5–100	2.9	[51]
GCE/GA-PEI	AA UA	DPV DPV	100–1000 10 -150	0.25 9	This work

*Acronyms of sensing platform in Supplementary information

## Conclusions

This work describes the modification of a glassy carbon electrode with an optimized mixture of graphene aerogel and PEI for quantifying uric acid and ascorbic acid. Detection of both analytes was enhanced individually and in mixtures, including in pharmaceutical samples for ascorbic acid and urine samples for uric acid. The electrochemical sensor achieved a limit of detection of 0.25 µM for ascorbic acid and 9 µM for uric acid. The GCE/GA-PEI platform is a versatile and promising analytical tool that enables new possibilities for developing enzyme-free electrochemical sensors, improves stability, and broadens the applications of these devices.

## Supplementary Information

Below is the link to the electronic supplementary material.

Supplementary Material 1

## Data Availability

Data are available on reasonable requests.
