# Supplementary material for: Dual electrochemical sensor based on graphene aerogel for quantification of ascorbic acid in pharmaceutical samples and uric acid in urine
Source: Anal Sci. 2026 Apr 15;42(6):365–76. doi: 10.1007/s44211-026-00896-z (PMC13201294; doi:10.1007/s44211-026-00896-z)
Supplement: Supplementary file 1 — Supplementary Material 1 [file 44211_2026_896_MOESM1_ESM.docx]

**Supplementary Information**

**Dual electrochemical sensor based on graphene aerogel for quantification of ascorbic acid in pharmaceutical samples and uric acid in urine**

Alejandro Gutiérrez^1^, Mario Sánchez-Suárez^2^, Natalia Rey-Raap^2^, Ana Arenillas^2+^, Janet Ledesma-García^1*^, Luis Gerardo Arriaga ^3^

^1^ División de Investigación y Posgrado, Facultad de Ingeniería, Universidad Autónoma de Querétaro, 76010, Santiago de Querétaro, México.

^2^ INCAR-CSIC, Instituto de Ciencia y Tecnología del Carbono, Francisco Pintado Fe 26, 33011, Oviedo, Spain.

^3^ Tecnológico de Monterrey, Institute of Advanced Materials for Sustainable Manufacturing, 76130, Santiago de Querétaro, México.

Corresponding authors: * [janet.ledesma@uaq.mx](mailto:janet.ledesma@uaq.mx); ^+^ [ana.arenillas@csic.es](mailto:ana.arenillas@csic.es)


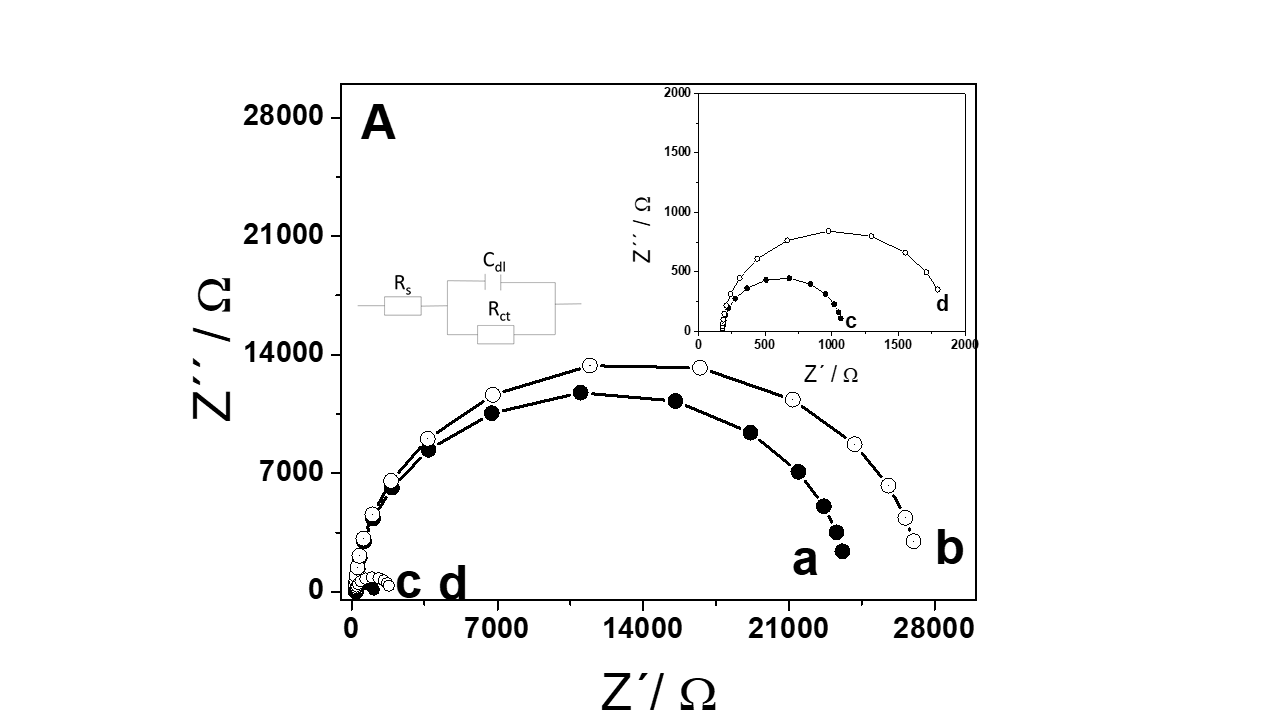


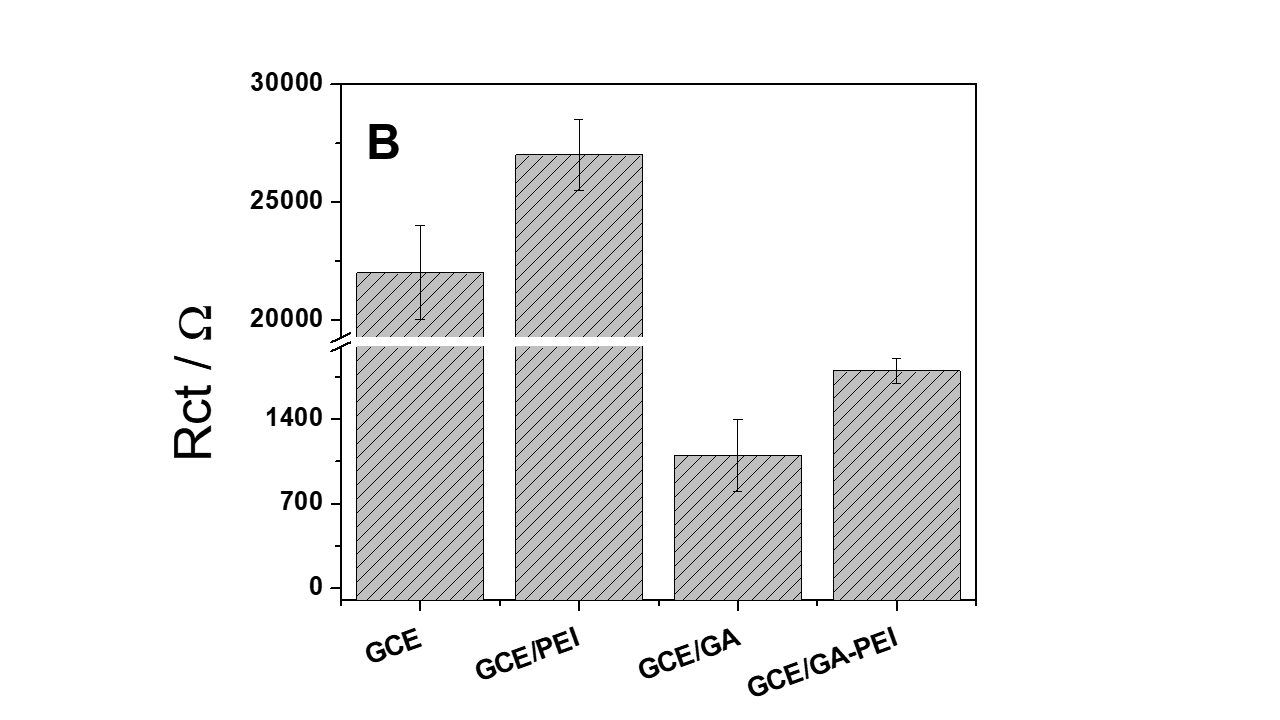


Figure S1. (A) Nyquist plots in the presence of 2.5 × 10⁻² M H₂O₂ obtained at (a) GCE, (b) GCE/PEI, (c) GCE/GA, and (d) GCE/GA-PEI. Working potential: 0.700 V. Frequency range: 10 kHz to 10 mHz; potential perturbation: 10 mV; supporting electrolyte: 0.050 M phosphate buffer solution, pH 7.40. Inset in Figure S1-A: Equivalent circuit used to fit the experiment, shown on a more sensitive scale. (B) Bar graphs of charge transfer resistance (Rct) obtained from the spectra shown in Figure S1-A. The error bars indicate the standard error of the mean (n = 3 electrodes).


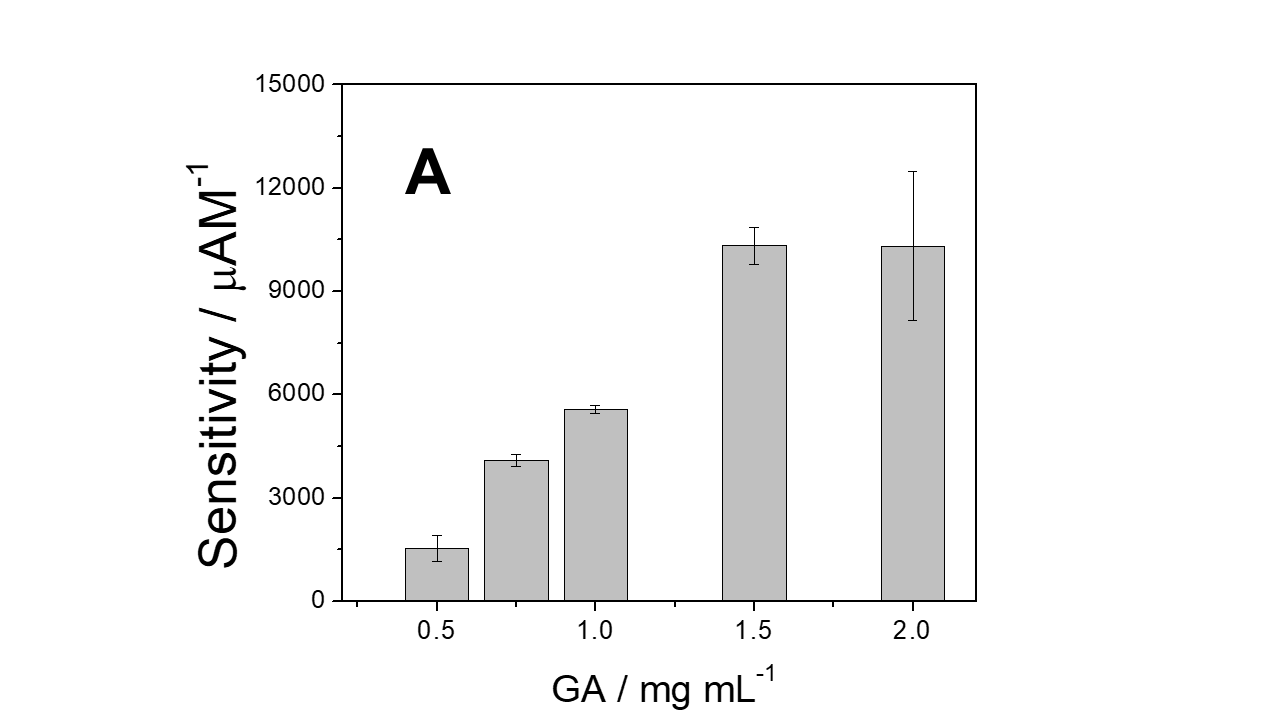


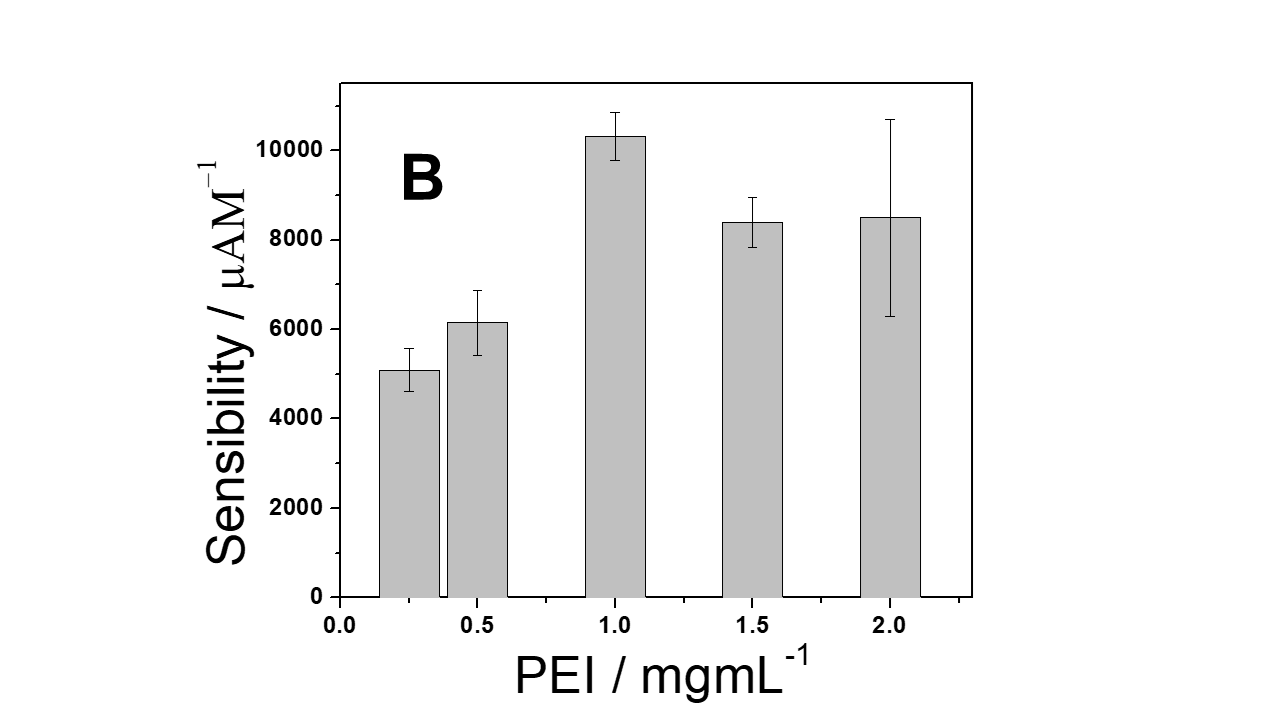


Figure S2. Variation in sensitivity to hydrogen peroxide from amperometric experiments at 0.700 V using (A) GCE modified with GA-PEI dispersions prepared by sonication with 1.0 mg mL⁻¹ PEI and varying amounts of GA; (B) GCEs modified with GA-PEI dispersions prepared by 15 minutes of sonication with 1.5 mg mL⁻¹ GA and different concentrations of PEI. Error bars represent the standard error of the mean (n = 3 electrodes).


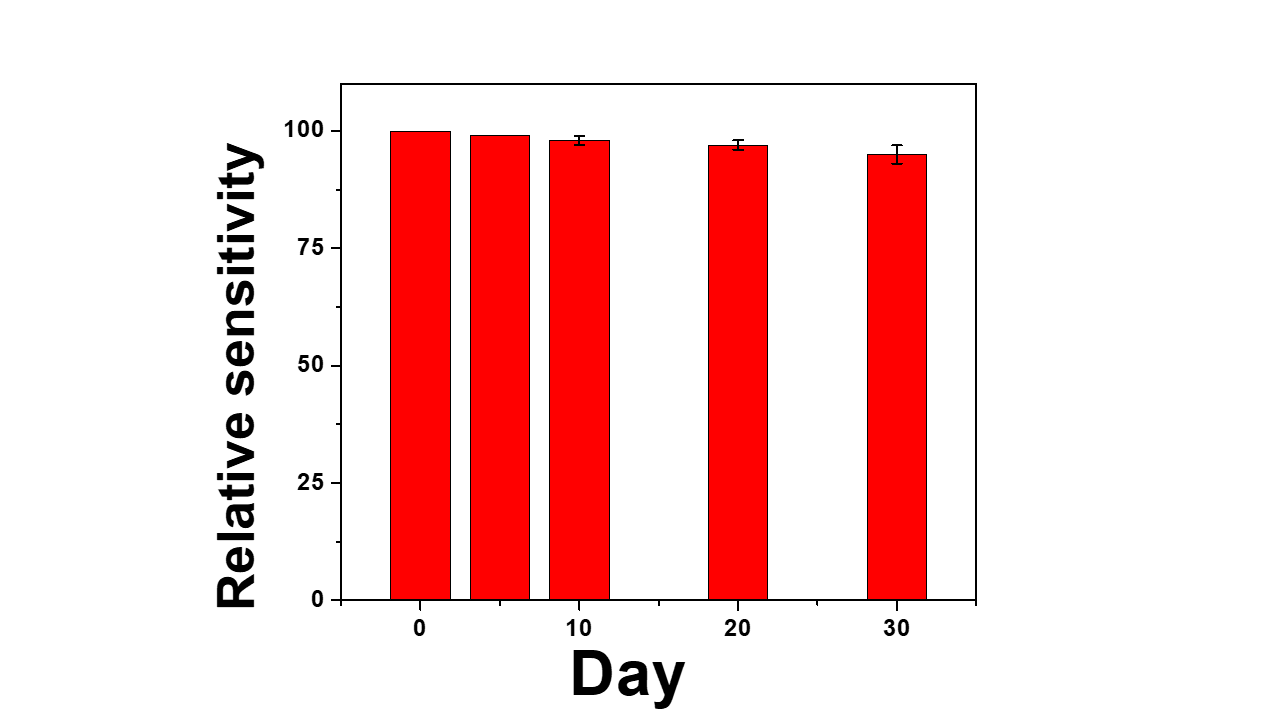


Figure S3. Relative sensitivity to hydrogen peroxide from amperometric experiments on GCEs prepared with the same GA-PEI dispersion stored at room temperature for different durations. Relative sensitivity is defined as S/So × 100, where S is the sensitivity obtained on a given day and So is the sensitivity obtained on the first day of the sensor. The error bars represent the standard error of the mean (n = 3 electrodes).


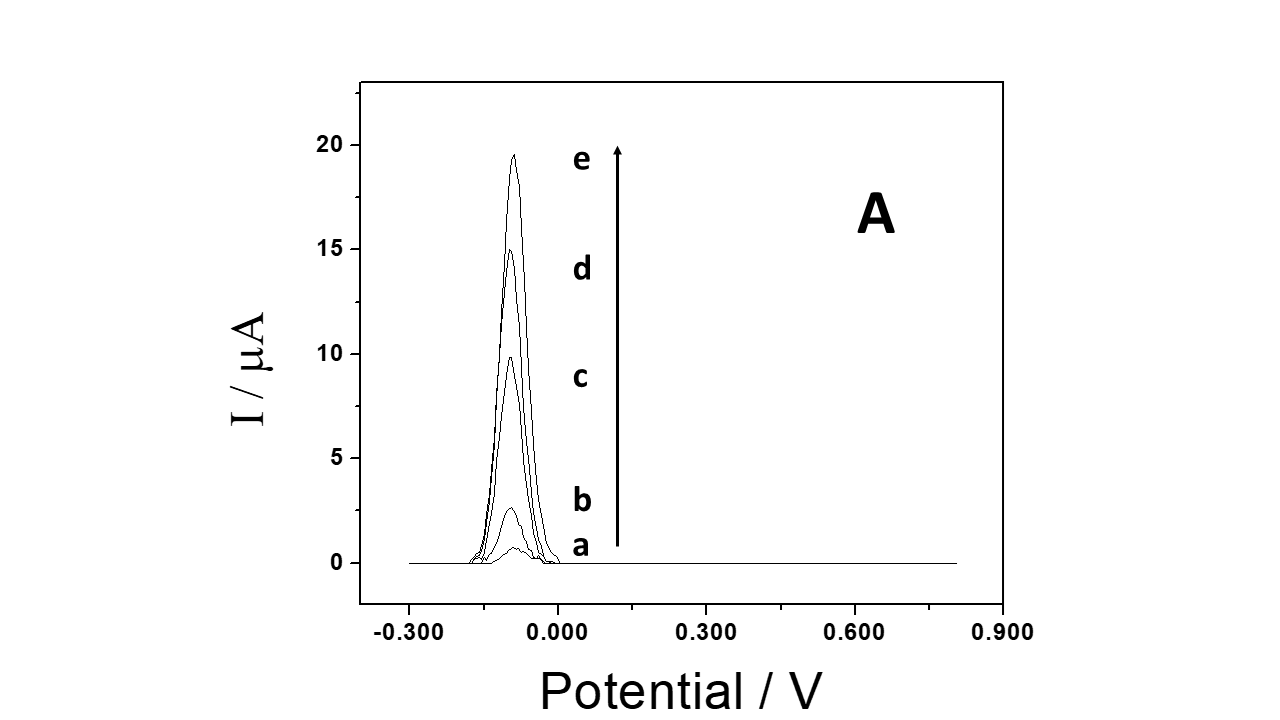


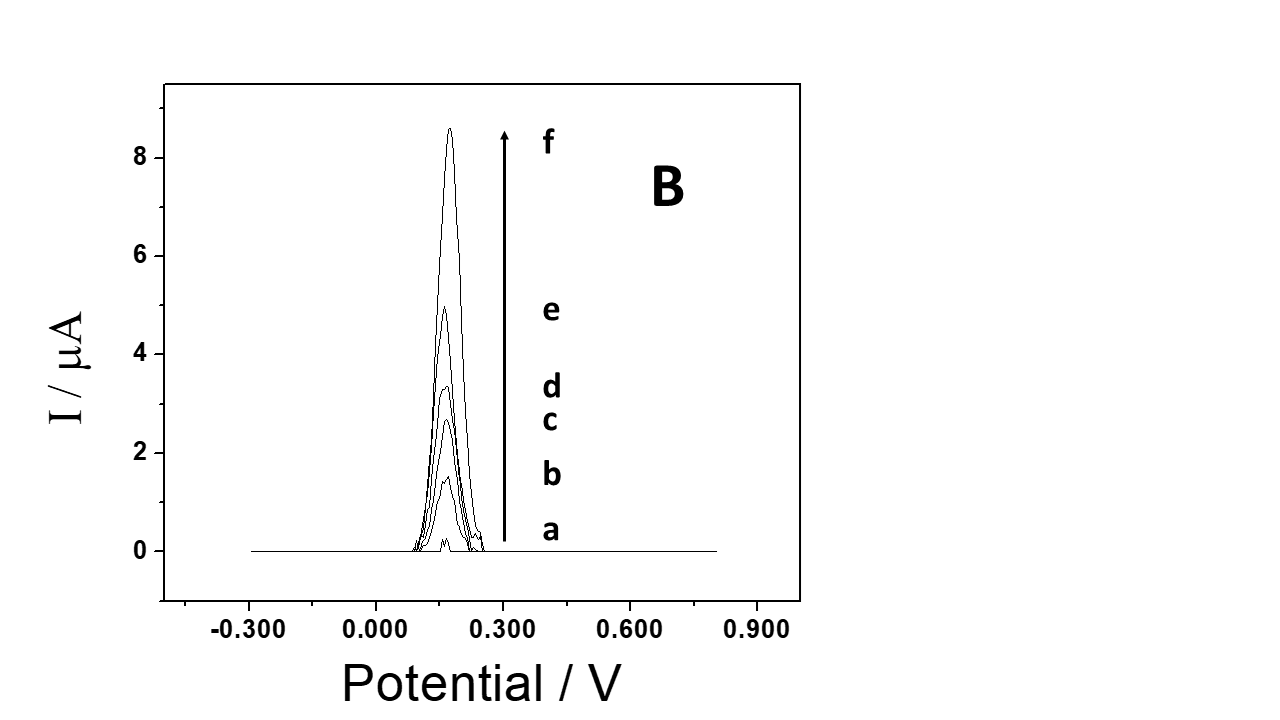


Figure S4. DPVs obtained in 0.050 M phosphate buffer solution at pH 7.40 with GCE/GA-PEI and AA (A): (a) 1.0 × 10⁻⁴ M, (b) 2.0 × 10⁻⁴ M, (c) 5.0 × 10⁻⁴ M, (d) 7.0 × 10⁻⁴ M, and (e) 1.0 × 10⁻³ M; or UA (B): (a) 1.0 × 10⁻⁵ M, (b) 3.0 × 10⁻⁵ M, (c) 5.0 × 10⁻⁵ M, (d) 8.0 × 10⁻⁵ M, (e) 1.0 × 10⁻⁴ M, and (f) 1.5 × 10⁻⁴ M.

Acronyms of sensing platform showed in Table 3.

N-doped graphene Aerogel: nitrogen-doped graphene aerogel; PEDOT:PSS/PVA/AuNPs/SPCE-PEDOT:PSS: Poly(3,4-ethylenedioxythiophene) polystyrene sulfonate; PVA: Polyvinyl alcohol; AuNPs: Gold nanoparticles; γ-Ni(OH)_2_–NiO(OH)/PANI-CNTs- Ni(OH)2–NiO(OH); nanoelectrocatalyst decorated on polyaniline–carbon nanotubes (PANI-CNTs) nanocomposite film; CUST-636: new Keggin-type POMOFs [Cd(btbu)_2_(H_2_O)_2_(PMo_12_O_40_)]; CUST-638: [Cd(btbu)_2_(H_2_O)(SiW_12_O_40_)_0.5_]; btbu: 1,4-bis(1,2,4-triazol-1-yl)butane; TMB-Pt@Ag NFs/uricase: 3,3´,5,5´-tetramethylbenzidine- Pt@Ag nanoflowers; MWCNT-polyArg: dispersion multi-walled carbon nanotubes and polyarginine; WB-S La^3+^/TiO_2_- NS/SPE: Woolen ball-shaped La3+/TiO2 nanostructure / screen-printed electrode; Pd-Co / UOx: Pd-Co aerogel / urato oxidase; Ni-Co / UOx: Ni-Co aerogel / urato oxidase. SWV- Square wave voltammetry; HPCE- high Performance capillary electrophoresis; SERS- surface-enhanced Raman scattering; DPV-Differential pulse voltammetry, LIG: laser-induced graphene.
